# Evaluating Ecohydrological Model Sensitivity to Input Variability with an Information-Theory-Based Approach

**DOI:** 10.3390/e24070994

**Published:** 2022-07-18

**Authors:** Mozhgan A. Farahani, Alireza Vahid, Allison E. Goodwell

**Affiliations:** 1 Department of Civil Engineering, University of Colorado Denver, Denver, CO 80204, USA; mozhgan.askarzadeh@ucdenver.edu; 2 Department of Electrical Engineering, University of Colorado Denver, Denver, CO 80204, USA; alireza.vahid@ucdenver.edu

**Keywords:** sensitivity analysis, quantization, information theory, rate-distortion theory, ecohydrological modeling

## Abstract

Ecohydrological models vary in their sensitivity to forcing data and use available information to different extents. We focus on the impact of forcing precision on ecohydrological model behavior particularly by quantizing, or binning, time-series forcing variables. We use rate-distortion theory to quantize time-series forcing variables to different precisions. We evaluate the effect of different combinations of quantized shortwave radiation, air temperature, vapor pressure deficit, and wind speed on simulated heat and carbon fluxes for a multi-layer canopy model, which is forced and validated with eddy covariance flux tower observation data. We find that the model is more sensitive to radiation than meteorological forcing input, but model responses also vary with seasonal conditions and different combinations of quantized inputs. While any level of quantization impacts carbon flux similarly, specific levels of quantization influence heat fluxes to different degrees. This study introduces a method to optimally simplify forcing time series, often without significantly decreasing model performance, and could be applied within a sensitivity analysis framework to better understand how models use available information.

## 1. Introduction

Ecohydrological models predict plant carbon uptake and surface energy partitioning that result from the biochemical and ecophysiological functioning of leaves and the structural components of the canopy. However, multiple model parameters, input forcings, boundary and initial conditions lead to high complexity and challenges to separate and understand different aspects of the ecosystem [1]. These features are typically uncertain, and it is therefore important to take these uncertainties into account. As such, techniques to evaluate the impacts of these uncertainties and the role of validation in process-based models has been extensively recognized [2,3,4,5,6,7,8], and a variety of approaches and tools for *Sensitivity Analysis (SA)* have been developed. *SA* is a procedure used to explore uncertainty and better understand how a complex model responds to varying inputs and parameters [9]. *SA* methods consider different individual or multiple working hypotheses which involve input data sets, parameters sets, model structures, and process equations [10,11]. Specifically, *SA* uses the entire allowable ranges of model inputs and evaluates how model uncertainty can be apportioned to the uncertainties in model inputs [12]. Sensitivity tests are useful experimental tools for hypothesis testing and model evaluation and are employed to improve and calibrate model predictions [13,14].

Many ecohydrologic modeling studies have focused on model sensitivity to the modeling hypotheses (e.g., parameter specification [15,16,17] and model structures [11,18]). Here, we consider a model that is constant in terms of structure, processes, and parameters such that the information provided by these modeling hypotheses is conditional on the nature of the input forcing data and their associated uncertainties [19,20,21]. It has been hypothesized that the mean interannual total sensitivity indices of forcing error (or input data error) is greater than that of the model structure (or process representation) and parameter choice in a multiphysics snowpack model [22]. In other words, errors in forcing and validation data are the greatest factors affecting the model performance [23]. For example, Essery et al. [24] presented a comparison of the forcing error uncertainty to model structure uncertainty originating from 1701 model structures.They found that even forcing scenarios with moderate precipitation errors have a larger influence on snowpack simulation outputs than different model structures at one site. Raleigh et al. [25] examined various scenarios of forcing data errors and their associated uncertainty in snow models. They identified the relative significance of individual forcing variables on snow model output variance using a *SA* framework and found a large impact of forcing biases on snowpack simulations over different output metrics. While these studies quantify the importance of high quality forcing variables, they do not address aspects of temporal variability or precision. Here, we focus on the response of a model’s predictions to changing forcing data precision to better understand how the model uses information from forcing data, particularly variability within forcing data. This aspect of forcing data precision in a complex ecohydrological model is typically dictated by the precision of measurement devices, which may vary significantly between data types and instruments.

The forcing variables needed to run models are prone to errors, especially when they are spatially interpolated from nearby climate sites or are provided by satellite-derived data. In general, the forcing data conditions and shapes the dynamical response of a system. No matter how well a model is formulated, if the forcing data, as obtained from in situ or remotely sensed observations, does not reflect existing causal mechanisms from inputs to observed outputs, the model cannot account for these mechanisms. We hypothesize that we can optimally minimize the complexity of a forcing dataset, without greatly changing model predictive performance or behavior. This hypothesis relates to Occam’s razor, which is a science philosophy that states that the preferred model is the simplest that produces a given level of predictive performance [26]. Meanwhile, we also expect that a model will be more sensitive to the precision of some forcing variables relative to others, and models may have nonlinear responses to changing the precision of multiple forcing variables. We first demonstrate an analytical method to optimally approximate representations of model input variables at lower precision and minimize the complexity of a forcing dataset while retaining its most relevant features. We retain the complexity of the model itself, and focus purely on the effect of forcing data complexity. Decreasing precision or size of forcing data results in less memory storage needed and minimizes impacts of instrument noise in the forcing dataset, which are desirable features. Additionally, manipulating the precision of forcing data can help identify model sensitivities and potential structural errors to the extent that a model does not take advantage of the full precision of the available input datasets. For example, if a decrease in the precision of a forcing variable weakens the apparent source–target relationship in observed data but does not lead to a similar change in model behavior, this would indicate the model is not utilizing the level of detail available in the original forcing data. We address the following relevant questions: *How does a model respond to the precision and variability of forcing data? Specifically, how does decreasing precision of individual or multiple forcing variables affect model sensitivity?* From an ecohydrologic modeling perspective, specific questions could include: *How does a model respond to changes in combinations of meteorological forcing inputs relative to inputs that are manipulated one at a time? For a model with multiple land–atmosphere flux outputs, to what extent are some fluxes responding to forcing precision or utilizing available information differently from others?* To answer these questions, we use rate-distortion theory, a branch of information theory.

Information Theory (IT) [27] provides the theoretical and fundamental limits of data compression, representation, and transmission. IT methods have been used in Earth science studies to construct ecohydrologic process networks that reveal ecosystem behaviors [11,28,29,30,31,32,33,34] and analyze models and related hypotheses [13,35,36,37]. Input variables are not usually represented as continuous since measurement instruments typically record data discretely to some degree of precision. Therefore, discrete representation of continuously varying quantities highlights that no data is infinitely precise [38]. This makes it useful to define the “goodness” of a representation of data. We use rate-distortion theory, a branch of IT, to optimally approximate representations of model input variables at different precisions. Specifically, we quantize input variables to different precisions in a way that minimizes the distortion at a given precision. This is accomplished by defining a distortion measure, which is a measure of distance between a random variable and its representation [39]. We use this method to analyze the sensitivity of an ecohydrological model to precision in different forcing variables. This paper is organized as follows. In Section 2, we provide an overview of the model background and characteristics of the forcing data. Then, we provide background on quantization and introduce our approach to quantize forcing data and evaluate model results. In Section 3, we discuss results, and in Section 4, we present discussion and conclusions.

## 2. Materials and Methods

### 2.1. Multi-Layer Canopy Model and Forcing Data

We study model sensitivity to input forcing variability using MLCan [40,41] as an example of a complex ecohydrological model that uses many high-resolution meteorological inputs as forcing data. MLCan is a multi-layer canopy–soil–root model that vertically resolves the canopy radiation, meteorological micro-environment, biochemical process, and leaf level ecophysiological states at multiple canopy levels to simulate net canopy--atmosphere fluxes of carbon dioxide (*Fc*), latent heat flux (*LE*), and sensible heat flux (*SH*). There is a flexibility in the choice of photosynthetic pathway of agricultural crops, soybean (*C3*) or maize (*C4*).

Previous sensitivity studies of MLCan have focused on the resolution of canopy and soil layers, initial conditions, and parameter uncertainty. Drewry et al. [40] examined the effects of vertical canopy resolution, or the number of layers used to subdivide the canopy for maize and soybean crop types, on model estimates of canopy radiation regime, leaf states, and canopy--atmosphere fluxes. They also analyzed the impact of meteorological conditions in controlling the flux variation and examined the role of important environmental drivers (*Rg*, *Ta*, *VPD*, and *U*) and modeled skills for capturing variability in canopy-scale exchange of $CO_{2}$ and latent and sensible heat for maize and soybean. Recently, Ruddell et al. [11] examined one-at-a-time parameter sensitivity with a focus on the stomatal slope parameter in the Ball--Berry model [42] which relates the scaling of the stomatal function to photosynthesis and ambient conditions at the leaf surface to the model’s three key outputs of carbon and energy fluxes (*Fc*, *LE*, and *SH*).

We ran the model over a 100-day period (DOY 150-250) of the 2018 growing season using meteorological forcing data from a 25 m flux tower located at Goose Creek, Central Illinois (https://imlczo.ncsa.illinois.edu (accessed on: 8 June 2022)).This site is part of the Critical Zone Interface Network (CINet) project, formerly the Intensively Managed Landscape Critical Zone Observatory (IMLCZO). These forcing data include 15 min shortwave (*Rg*), canopy-top air temperature (*Ta*), vapor pressure deficit (*VPD*), ambient $CO_{2}$ concentration (*Ca*), wind speed (*U*), air pressure (*Pa*), and precipitation (*PPT*). In addition to filling the flux tower data gaps using REddyProc eddy covariance data processing (https://www.bgc-jena.mpg.de/bgi/index.php/Services/REddyProcWebRPackage (accessed on: 8 June 2022)), extreme outliers were removed.

The 25 m flux tower observes a mosaic of maize and soybean fields within its shifting flux footprint. This flux footprint is the contributing area to a given flux measurement and varies with wind direction, wind speed, surface roughness, and atmospheric conditions [43]. We ran MLCan for both maize and soybean vegetation types and applied a flux footprint model to represent their flux contributions at each time step. Particularly, the relative contribution of each crop type to these measured fluxes within the flux tower footprint was considered based on a 1D flux footprint model [44] and a USDA land-use map that distinguishes between maize and soybean fields for a growing season (https://nassgeodata.gmu.edu/CropScape/ (accessed on: 8 June 2022)).The flux footprint model provides estimated fractions of momentum flux from each crop, and we estimated the modeled flux (*X*, where *X* is *Fc*, *LE*, or *SH*) as follows: $X=f_{m}X_{m,MLCan}+f_{s}X_{s,MLCan}$. Here, $f_{m}$ and $f_{s}=1-f_{m}$ are contributing fractions of maize and soybean fields, respectively, according to the flux footprint model, and $X_{m,\mathrm{MLCan}}$ and $X_{s,\mathrm{MLCan}}$ are the MLCan simulated fluxes for each crop type. In this way, the modeled flux at a given time step is a weighted average of the modeled flux based on two crop types. While this has implications for the overall fluxes measured at the tower [45], we find that in terms of variability, since both maize and soybean simulations are based on the same input data, this averaging has a minor impact on the links between forcing precision and model outputs. In other words, changing forcing precision has a similar effect on model output regardless of vegetation photosynthesis pathway.

Here, we study the impact of the precision of several forcing variables on model outputs at an agricultural site as follows:

- Air temperature (*Ta*) affects stomatal conductance of plant and evaporation from both soil and canopy. The Campbell 083E instrument located at the flux tower has a precision for measuring *Ta* of $\pm 0.1{\;}^{\circ }$C.
- Wind speed (*U*) affects canopy evaporation and associated heat flux partitioning. The precision of the CSAT3 measurement instrument at the tower for *U* is ±0.1 m/s.
- Vapor pressure deficit (*VPD*) is the difference between saturation water vapor pressure (*es*) and actual water vapor pressure (*ea*). It is the measure of atmospheric desiccation strength [46] and indicates the atmospheric demand for water vapor. The *ea* is determined by a large number of drivers and is also related to *Ta* in that it is upper bounded by *es*.
- Shortwave radiation (*Rg*) is a principle driver of $CO_{2}$, vapor, and heat exchange available in the system which controls photosynthesis and leaf energy balance.

We separate the analysis of the energy balance forcing variable *Rg* from the meteorological forcing variables, *Ta*, *U*, and *VPD*, into two case studies. First, *Rg* is quantized as an example where decreasing precision alters the energy balance, and we expect resulting changes in heat fluxes to reflect that in a uniform way. Meanwhile, other meteorological variables that do not affect the energy balance are used within the model in multiple processes, and the effect of changing precision may be more nuanced. We retain all other input forcing data, including air pressure, carbon concentrations, and vegetation indices, as their full distributions of values, with reasoning as follows:

- Precipitation (*PPT*) is often zero at the 15-min resolution considered here, such that changing the precision of *PPT* would not have any effect for most time steps.
- Carbon dioxide concentration (*Ca*) is a model parameter and is constant.
- Air pressure (*Pa*) varies within a very small range, and the model is relatively unresponsive to fluctuations in *Pa*. In other words, quantizing *Pa* would not lead to measurable differences in model behavior relative to other inputs.
- Canopy structure is described by leaf area density (*LAD*) profiles and the total leaf area index (*LAI*). In the model, the *LAI* is independent of all other forcing conditions and is based on surveys of maize or soybean plants throughout each year. It typically increases monotonically through the growing season and does not vary on a sub-daily to daily timescale.

### 2.2. Information Theory to Manipulate Forcing Precision

Shannon entropy is a measure of the uncertainty of a random time-series variable, which is computed based on its probability distribution function. It is the expected number of yes–no questions (the amount of information) in units of “bits” needed to correctly determine an occurrence of the event. If a continuous variable *X* is represented as a discrete variable $X_{d}$ by binning the continuous values into $N_{d}$ bins centered at the discrete values $\left\{x_{1},\ldots ,x_{N_{d}}\right\}$, Equation (1) defines the discrete entropy with bin-width *D* [27]:

(1) $$H\left(X_{d}\right)=-\sum_{n=1}^{N_{d}}P\left(x_{n}\right)\log P\left(x_{n}\right),$$

where *n* is the bin number, $N_{d}$ is the number of bins, and $P(x_{n})$ is the proportion of data falling into the *n*th populated bin which is the probability that $X_{d}=x_{n}$. Since entropy is a function of the distribution of *X*, it does not depend on the actual values taken by the random variable *X* but only on the probabilities. Therefore, we can use entropy as a way to compare the distributions based on different ways to discretize the data and the maximum possible entropy which corresponds to a uniform probability distribution where every bin is populated with an equal number of data. According to Equation (1), $P\left(x_{n}\right)=1/N_{d}$ for all *n* where $N_{d}$ is the total number of bins and this gives: $H_{max}=\log_{2}\left(N_{d}\right)$.

Discretization occurs when we measure values for continuous variables with some level of precision, rounding up or down to the nearest finite precision value available to the measuring instrument. Since exact representation of a random variable requires infinite precision, we cannot reproduce it exactly using finite information bits. Rate-distortion theory quantifies the trade-off between the reconstruction quality and the number of bits used for data representation [39]. In the context of the current work, our flux tower forcing dataset is already quantized to the extent that the instruments collect data at a certain high precision.

#### 2.2.1. Quantization

Quantization is a data processing technique that takes a large range of values and represents them by a (much) smaller set of values [47]. Data binning is a form of quantization in which the variables that fall into a bin are replaced by a value representative of that bin ($\hat{X}$), often the central value. In this proposed method, we also need a probability distribution which is a function that characterizes how a random variable is selected from among the possible values it may obtain. In this regard, the oldest and most widely used approach of estimating a distribution is bin counting (or histogram). Suppose that $x_{0}$ is a given value, and δ is the width of the bin containing data values. The *i*th bin of the histogram is defined as $\left[x_{0}+i\delta ,x_{0}+\left(i+1\right)\delta \right]$, and a count of points in this range represents the probability that a data value falls within the *i*th bin. The estimated distribution $\hat{f}\left(x\right)$ [39] is given by Equation (2):

(2) $$\hat{f}\left(x\right)=\frac{c_{x_{i}}}{n\delta }\;\mathrm{for}\;x\in \left[x_{0}+ih,x_{0}+\left(i+1\right)h\right],\;i=1,\cdots ,N_{b}$$

where $c_{x_{i}}$ is the count of the data values that fall into the *i*th bin, and *n* is the total number of data values. The bin width parameter (δ) has a significant effect on the robustness and accuracy of the estimated distribution. In this study, we estimated the width of our initial histogram bins using the “normal reference rule” [48] where the optimal width varies with each input variable based on its variance assuming that the data conform to a normal distribution (Table 1).

**Figure 1:**
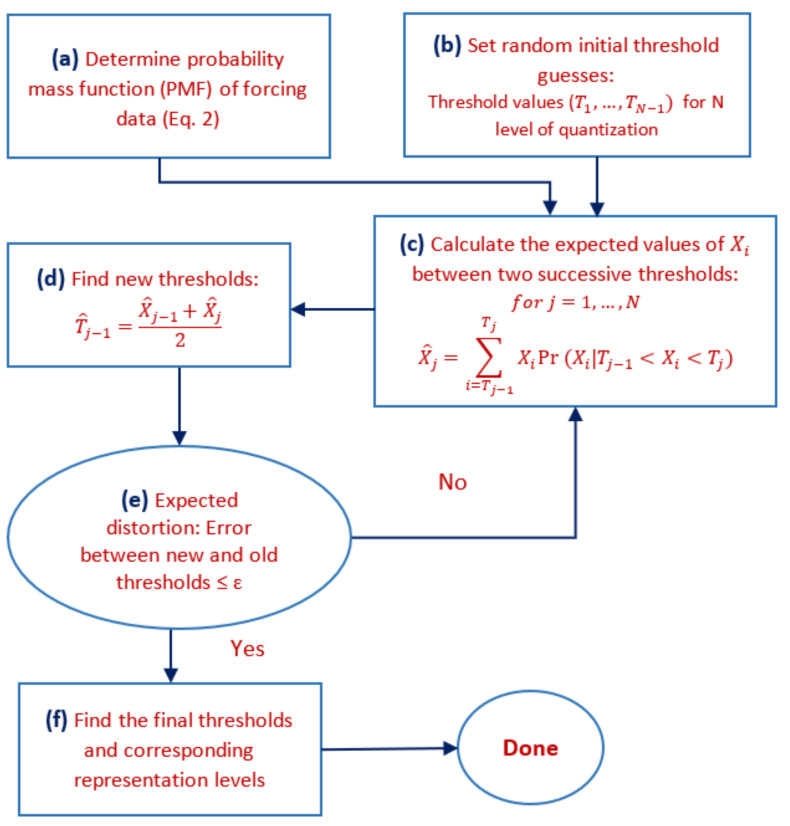
Flowchart of Lloyd algorithm used for quantization of model forcing variables.

After finding the distribution of input variables, we used the Lloyd [49] algorithm to quantize input variables to *N* levels, where *N* is set to {2,3,4,5} (Figure 1). We quantize the input variable to the lowest precision of N=2 and increase it to 5 to show the model responds to different low precisions and variability of forcing data. We assumed that the full precision of the input datasets is the original forcing dataset. This algorithm begins with a random initial “guess” of where thresholds should lie (Figure 1b), and the representation points are defined as the expected value of a point within that threshold (Figure 1c). Then we find the optimal thresholds points within these thresholds and repeat the iteration for this new set of threshold points. The expected distortion is decreased at each stage in the algorithm so the algorithm will converge to a local minimum of the distortion. The goal of the algorithm is to minimize the distortion, which is a measure of the cost of representing the variable *X* by its quantized representation point. We note that there are various distortion metrics used for different applications, but minimum mean square error (MMSE) is commonly used for natural data. Moreover, the benefits of rate-distortion theory go beyond discretizing continuous data to minimize a given distortion metric. In fact, it has been shown that once the quantization bins are determined, it might benefit the overall system to induce controlled distortion. For example, adding controlled distortion would enable the creation of many more data representatives, which is of value in data storage and communications [50,51,52,53]. Therefore, an *N*-level quantizer *Q* can be characterized in terms of a set of *N* real-valued quantization points. This process is summarized in Figure 1 and in the following paragraphs along with an example of its application to air temperature, *Ta*, in the next subsection.

#### 2.2.2. Illustrative Example of Quantization: Air Temperature

We start by finding the probability mass function of the *Ta* dataset (Figure 1a). Then, N−1 random initial threshold guesses for *N* levels of quantization are set (Figure 1b), denoted as ($T_{1},\cdots ,T_{N-1}$). These may be any values between the minimum value ($T_{0}=X_{min}$) and the maximum value of data ($T_{N}=X_{max}$). Next, based on estimated PMF, the expected value of the data between each pair of successive thresholds is calculated [39] as follows (Figure 1c):

(3) $${\hat{X}}_{j}=\sum_{i=T_{j-1}}^{T_{j}}X_{i}Pr\left(X_{i}|T_{j-1}<X_{i}<T_{j}\right),\;\mathrm{for}\;j=1,\cdots ,N$$

From this, we obtain corresponding representation points (${\hat{X}}_{1},{\hat{X}}_{2},\cdots ,{\hat{X}}_{N}$) for each input variable. For example, for our *Ta* dataset for N=2, the randomly selected initial threshold guess is $T_{1}=19.15{\;}^{\circ }$C, which leads to two representation points of ${\hat{X}}_{1}=17.3{\;}^{\circ }$C and ${\hat{X}}_{2}=24.2{\;}^{\circ }$C.

Based on the representation points that correspond to our initial threshold guesses, we next update the threshold values as the midpoints between two successive representation points, with a goal to decrease the distortion, as follows (Figure 1d):

(4) $${\hat{T}}_{j-1}=\frac{{\hat{X}}_{j-1}+{\hat{X}}_{j}}{2}$$

For example, for *Ta*, the new threshold is computed as $20.77{\;}^{\circ }$C. In the Lloyd algorithm, the quality of quantization can be measured by the distortion of the resulting representation in comparison to the original values. The distortion function $d(X,\hat{X})$ is a measure of the cost of representing the *X* by $\hat{X}$. We use the expected squared-error distortion [39], which is a commonly used distortion function (Figure 1e):

(5) $$d\left(X,\hat{X}\right)=E{\left(X-\hat{X}\right)}^{2}$$

**Figure 2:**
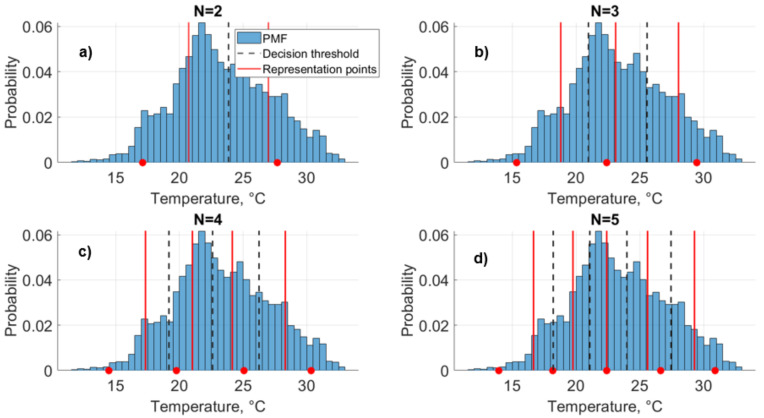
(**a**–**d**) Illustration of rate-distortion theory applied to air temperature (*Ta*) to quantize the data to different precisions. Red lines indicate the representation points and dashed lines indicate final decision thresholds for $N_{T}=2,\cdots ,5$ levels of quantization for *Ta* in the growing season (DOY 150-250). The blue bars indicate the underlying probability mass function of *Ta*. Representation points alternately calculated with the fixed binning method are shown with red dots.

**Figure 3:**
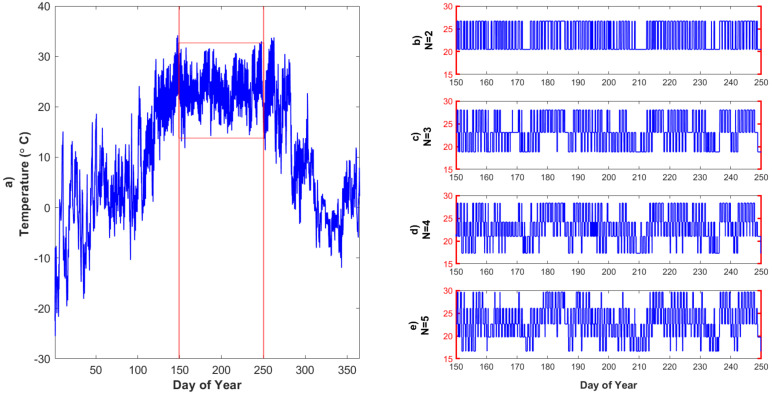
The original *Ta* forcing data and quantized forcing data for the growing season based on the Lloyd algorithm: (**a**) original *Ta* time-series data, where red lines indicate start and end of the peak growing season (DOY 150-250); (**b**) simplified *Ta* time- series data with $N_{T}=2$ levels of quantization, (**c**), $N_{T}=3$ levels of quantization, (**d**) $N_{T}=4$ levels of quantization, and (**e**) $N_{T}=5$ levels of quantization.

In the *Ta* dataset, this distortion measure is $1.62{\;}^{\circ }$C based on our initial threshold guesses. The Lloyd algorithm iterates through this process, and distortion decreases at each iteration until the thresholds converge within a small tolerance, and the final decision threshold and corresponding representation points are determined (Figure 1f). Decision thresholds are used to define the intervals for the quantizer, $\alpha_{j}=\left(T_{j-1},T_{j}\right],\;for\;j=1,2,\cdots ,N$. For a given data value as $X\in \alpha_{j}$, that value is quantized into the point ${\hat{X}}_{j}$. For the *Ta* example, for N=2, $\alpha_{1}=\left[T_{0},T_{1}\right]=\left[11.8,23.6\right]{\;}^{\circ }$C and $\alpha_{2}=\left[T_{1},T_{2}\right]=\left[23.6,33\right]{\;}^{\circ }$C after 7 iterations (Figure 2a). In other words, the final threshold is $T_{1}=23.6{\;}^{\circ }$C. Therefore, for a given data value as $X\in \alpha_{1}$, that value is quantized into the representation point ${\hat{Ta}}_{1}=20.5{\;}^{\circ }$C and as $X\in \alpha_{2}$, that value is quantized into the representation point ${\hat{Ta}}_{2}=26.7{\;}^{\circ }$C. Based on this approach, we simplified individual forcing time-series data to N=2 levels of quantization (Figure 3b).

Therefore, we can find the representation points and final decision thresholds for $N_{T}=2,\cdots ,5$ levels of quantization for *Ta* in the growing season (DOY 150-250) in order to simplify temperature forcing data to different precisions (Figure 3) such that there are only {2,3,4,5} representation points. As *N* increases, the forcing data approaches the initial input data.

For a comparison, the difference between representation points for different precisions for *Ta* is calculated with the fixed binning method and the Lloyd algorithm (Figure 2). In fixed binning, the input variable range, the difference between maximum and minimum values, is equally divided to *N* bins. The center value of each bin is the corresponding representation point. This example illustrates how the Lloyd algorithm is applied to any variables and how quantized representation points can vary significantly between it and fixed binning method.

#### 2.2.3. Quantized Model Cases

**Figure 4:**
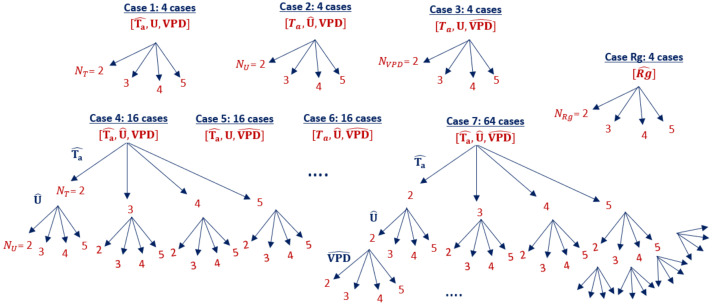
Model cases of simplified forcing data formed on different individual or joint quantization of the input variables (indicated by ^ symbol) and levels of quantization (N∈[2,3,4,5]). $\hat{Ta}=$ quantized air temperature to $N_{T}$ levels, $\hat{U}=$ quantized wind speed to $N_{U}$ levels, $\hat{VPD}=$ quantized vapor pressure deficit to $N_{VPD}$ levels and $\hat{Rg}=$ quantized shortwave radiation to $N_{Rg}$ levels.

We define seven model cases based on individual or joint quantization of the meteorological forcing variables, *Ta*, *U*, and *VPD*, and a single model case in which the energy balance forcing variable *Rg* is quantized as an example where quantization alters the energy balance. Since Rg is nearly zero in the forcing data at nighttime, Rg is only quantized for daytime points. Each case has a different simplified forcing dataset corresponding to a level of quantization. Based on how many forcing variables are quantized (f∈[1,2,3]), each case consists of $4^{f}$ different simplified forcing datasets. For example, Case 4 [$\hat{\mathrm{Ta}},\hat{U},\mathrm{VPD}$] has 16 different simplified forcing data sets, since ${\hat{T}}_{a}$ and $\hat{U}$ values are quantized to 2,⋯,5 levels and *VPD* is not quantized. Meanwhile, in Case 7 [$\hat{\mathrm{Ta}},\hat{U},\hat{\mathrm{VPD}}$], all three input variables are quantized to different levels such that there are 64 total forcing scenarios within this subset (Figure 4).

We use the simplified input forcing data for each forcing scenario within each case to estimate net canopy--atmosphere fluxes of carbon dioxide (*Fc*), latent heat flux (*LE*), and sensible heat flux (*SH*) using MLCan. This results in 124 model runs and quantized model results for {Ta,U,VPD} and 4 model runs and quantized model results for *Rg*. We refer to the model results based on the original forcing data as the “full” model and the model results running with any combination of quantized forcing data as a “quantized” model.

### 2.3. Sensitivity Analysis

We examine the impact of different precisions of forcing data on the model response by comparing the behavior of the quantized model results with both the full model results of *Fc*, *LE*, and *SH*. This comparison indicates the extent to which changing the precision of input variables alters the model behaviors. We identify the “sensitivity” of the model (SM_t_) to input variability by comparing the model response to changing input precisions individually and jointly. In this regard, we defined Equation (6) to calculate the diurnal root mean square errors (RMSE) between quantized and full model cases. Since this definition is the difference between the full and quantized model with an RMSE metric, it does not indicate an “error” but a “sensitivity”. Equation (6) defines SM_t_ for a given time of day and variable over the 100-day study window:

(6) $${SM}_{t}=\sqrt{\frac{\Sigma_{d=1}^{D}{\left({\mathrm{Fmodel}}_{d,t}-{\mathrm{Qmodel}}_{d,t}\right)}^{2}}{D}},$$

where D=100 is the number of the model simulation days, ${\mathrm{Fmodel}}_{d,t}$ is the full model result at a given 15-min time step (t=1,⋯,96) for each model simulation day (d=1,⋯,D), and ${\mathrm{Qmodel}}_{d,t}$ is the quantized model result at a given time step (*t*) for each model simulation day.

We also address how quantization decreases the complexity of forcing data in addition to altering model outputs to a certain extent. We defined Equation (7) to calculate the model complexity ($C_{m}$) as the entropy of the forcing data for a quantized model ($H_{Q}$) with quantization levels $N_{T}$, $N_{U}$, and $N_{VPD}$ for the input variables relative to that of the full model, or non-quantized forcing data, as follows:

(7) $$C_{m}=\frac{H({\hat{Ta}}_{N_{T}},{\hat{U}}_{N_{U}},{\hat{VPD}}_{N_{VPD}})}{H(Ta,U,VPD)}$$

**Figure 5:**
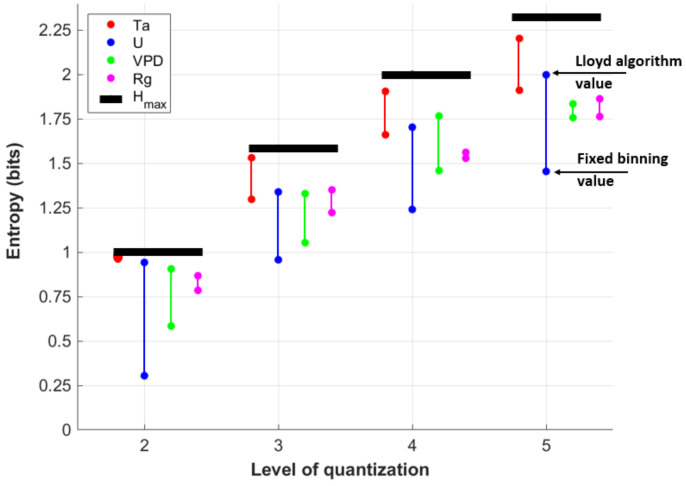
Differences between the entropy of the quantized forcing data of *Ta* (red dots), *U* (blue dots), *VPD* (green dots), and *Rg* (magenta dots) into *N* bins using fixed binning (lower points) and the Lloyd algorithm (higher points). A longer vertical line indicates that the Lloyd algorithm retains significantly more information relative to fixed binning. Black dots indicates maximum possible entropy ($H_{max}$) for each level of quantization based on a uniform distribution.

Here, we obtain a 3D bin distribution by dividing forcing variables *Ta*, *U*, and *VPD* into $N_{T}$, $N_{U}$, and $N_{VPD}$ bins each to measure complexity of the model at different forcing precisions. The number of bits needed for representing forcing data is calculated by entropy, which increases with more levels of quantization (e.g., Figure 5). If the forcing data have limited values and are skewed into a few bins (lower precision), the entropy of the dataset is low. Therefore, quantization decreases the complexity of data relative to the original distribution. Accordingly, $C_{m}$ is lower bounded by zero, corresponding to a distribution of data with “no” precision. $C_{m}$ is upper bounded by 1, corresponding to the full model case ($H{\left(Ta,U,VPD\right)}_{full}$) which is forced by the full distribution of data. The quantized model cases span the range of model complexities. For example, model case 7 ($\hat{\mathrm{Ta}},\hat{U},\hat{\mathrm{VPD}}$) for 2 levels of quantization for each variable leads to the lowest $C_{m}$ out of all cases.

## 3. Results

### 3.1. Effects of Quantizing Individual Forcing Variables

#### 3.1.1. Entropy

To explore the difference between two mentioned quantization methods, the fixed binning method and the Lloyd algorithm, we calculate the entropies of the four forcing variables at different levels of quantization. The entropy indicates how much information the forcing data contain. The difference between entropies for different precisions and quantization methods depends on forcing variable distribution. We find that the Lloyd algorithm always results in a higher entropy relative to fixed binning for a given quantization level *N* and a given variable type (Figure 5). In other words, the Lloyd algorithm results in a distribution that retains more information at a given quantization level. For reference, we compare these entropies at each quantization level to the maximum possible entropy ($H_{max}$, black lines in Figure 5). For forcing variables with a relatively normal distribution, such as *Ta*, we find that the entropy using the Lloyd algorithm is close to $H_{max}$, indicating that the threshold points are similar to quantiles of the data [54] in that there is a similar number of data points within each pair of threshold values. Meanwhile, the fixed binning entropy for *Ta* is also relatively high. For wind speed *U*, we see that the fixed binning entropies are very low, particularly for N=2, indicating a skewed or long-tailed distribution. Rate-distortion theory leads to a much higher entropy value, indicating that significantly more information is retained relative to fixed binning for a given *N*. Finally, *VPD* and *Rg* represent cases in between *U* and *Ta*, and there is less difference between fixed binning and the Lloyd algorithm representations of the data.

#### 3.1.2. Quantizing *Rg* Only: Changing Energy Balance Precision

**Figure 6:**
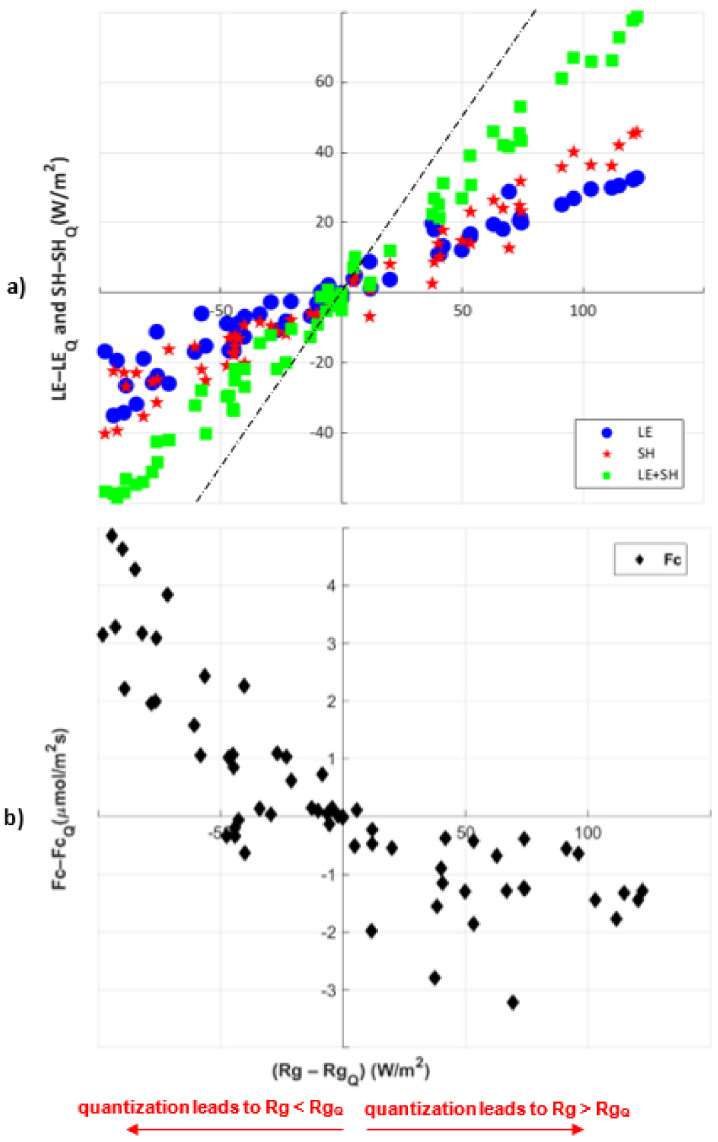
Quantized diurnal flux variation: (**a**) *LE* (blue circles), *SH* (red stars), and *LE+SH* (green squares); and (**b**) *Fc* (black diamonds) with quantized shortwave radiation *Rg* for N=2 level of quantization. X-axis indicates the difference between quantized shortwave radiation *Rg* and original forcing variable *Rg*. Y-axis indicates the difference between quantized model results and the full model results. The dashed line shows that the change in *Rg* is typically larger than *LE+SH*.

Since the heat fluxes, *LE* and *SH*, are constrained by available system energy, we find that changing radiation precision leads to corresponding changes in *LE* and *SH* (Figure 6a). Here, we only focus on the N=2 level of quantization for individual forcing variable, *Rg*, as the most extreme example of quantization. The difference between quantized *Rg* and the original forcing *Rg* is larger when *Rg* is maximum in the central part of the day, which corresponds to a larger distortion due to the quantization. The dashed line in Figure 6 shows that changes in *Rg* are typically larger in magnitude than the resulting *LE+SH* (green squares). In other words, a distorted *Rg* due to quantization leads to a slightly smaller joint distortion of *LE* and *SH* relative to the full model results. This distortion corresponds to changes in simulated ground heat flux (*G*) and longwave radiation that we do not consider here. In other words, a distorted *Rg* forcing input leads to a shift in all energy balance components. Otherwise, we see that differences between quantized and full model heat fluxes maintain a near-linear increase through the entire range of *Rg* variation, with some differences between *LE* and *SH*. Particularly, we find greater partitioning of energy to *SH* at the highest *Rg* distortion levels (Figure 6a). For example, if the N=2 level of quantization leads about *Rg-Rg_Q_* = 100 W/m${}^{2}$ for a given time point, this effect is not equally split between *LE* and *SH*. This difference in partitioning of energy is due in part to the effect of saturating net leaf carbon dioxide uptake (*An*) on stomatal conductance (*gs*) (Figure 6a) [42], which would impose an upper limit to *LE* and cause *SH* to increase to maintain the energy balance. We previously noted that due to the small amount of observed Rg in the forcing data during the night, *Rg* is not quantized at night.

The mean response of *Fc* to the quantized *Rg* for N=2 levels of quantization (Figure 6b) has different effects depending on whether quantization leads to an over- or under-estimation of *Rg*. When *Rg-Rg_Q_* is negative, we see larger changes in *Fc*, as *Fc-Fc_Q_*. This larger change means that when quantization leads to overestimating radiation input during the day (*Rg_Q_ > Rg*), the resulting carbon flux is lower (positive *Fc-Fc_Q_*). However, since daytime carbon fluxes are generally negative, this indicates that Fc_Q_ is actually larger in magnitude, and more photosynthesis is occurring. Similarly, when quantization leads to underestimating *Rg* input (*Rg > Rg_Q_*), we see that daytime *Fc-Fc_Q_* is negative, indicating that Fc_Q_ decreases in magnitude relative to the regular model but to a lesser extent. This behavior relates to soil carbon respiration and vegetation photosynthesis in the model. A change in radiation alters both soil respiration and photosynthesis, but this effect is nonlinear and depends on whether radiation is increased or decreased.

#### 3.1.3. Quantizing *Ta* Only: Temporally Varying Responses to Quantization

Next we focus on the effects of quantization of air temperature in Case 1 ($\hat{\mathrm{Ta}},U,\mathrm{VPD}$) of our seven cases that involve combinations of quantized air temperature, vapor pressure deficit, and wind speed. Case 1 has four different model input scenarios in which *Ta* is quantized for N=2,⋯,5 levels.

**Figure 7:**
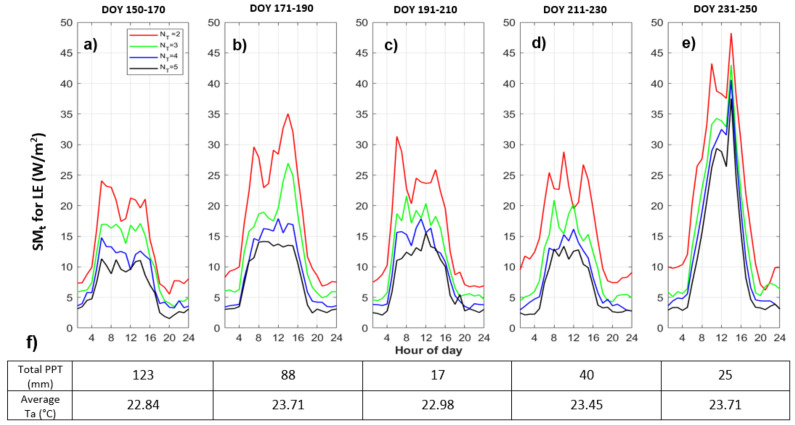
Quantized model sensitivity (SM_t_) for Case 1 ($\hat{\mathrm{Ta}},U,\mathrm{VPD}$) of *LE* (W/m${}^{2}$) within five 20-day time windows through the growing season: (**a**) for DOY 150-170; (**b**) for DOY 171-190; (**c**) for DOY 191-210; (**d**) for DOY 211-230; (**e**) for DOY 231-250; (**f**) the total PPT (mm) and the averaged *Ta* (${}^{\circ }$C) within given time window.

To illustrate how quantization effects model behaviors under different environmental and wetness conditions, we analyzed quantized model results for five 20-day windows during the 2018 growing season. Figure 7 shows the quantized model diurnal cycle sensitivity (SM_t_, Equation (6)) within each time window for *LE*. First, we note that the model response to levels of quantization is nonlinear. For example, during all time windows, $N_{T}=2$ levels of quantization leads to the most changes in the model, and the difference between $N_{T}=2$ and $N_{T}=3$ is usually greater than that between $N_{T}=4$ and $N_{T}=5$. This shows that quantization to a very few levels makes a larger difference relative to changing precision more slightly.

The first time window (DOY 150-170) is during the end of May until mid-June, which is earlier in the growing season, and water availability is higher than other time windows (more total precipitation, *PPT*). For this window (Figure 7a), the SM_t_ for the quantized cases are lower than for later times, which means the quantized and full models are more similar. The largest SM_t_ occurs in the last window (DOY 231-250) for $N_{T}=2$ levels of quantization. This time window, late in the growing season, is during mid-August until early-September which is relatively dry and warm and more water-limited relative to other time windows. During this time window, vegetation is very mature (highest *LAI*) which results in a large potential to uptake and release water. This large water potential causes a larger temperature dependency, such that the same quantization of *Ta* leads to a larger response in *LE* relative to earlier in the growing season, as *Ta* influences atmospheric demand for water and the ability to photosynthesize. In other words, when water is limited and there is high demand, the precision of *Ta* can have a large effect on heat fluxes. The largest SM_t_ occurs in the last window for $N_{T}=2$ levels of quantization. This largest difference between the quantized model and the full model occurs at midday (Figure 7e) when *Ta* is maximum. This is the time when the quantized *Ta* time series essentially switches from its “low” value at night to its “high” value during the day.

While the SM_t_ follows the diurnal cycle in that it increases with flux magnitudes, Figure 7 shows that nighttime values are also impacted by quantization, particularly for $N_{T}=2$ levels of quantization. For example, the ratios of nighttime to daytime SM_t_ value of *LE* (about 1:2) are a lot larger than the ratio of nighttime to daytime actual value of *LE* (about 1:10), meaning that nighttime values are actually affected disproportionately. This variability based on times of day and over different time windows through the growing season indicates that model sensitivity varies along with different thresholds and system states.

### 3.2. Model Sensitivity to Combinations of Forcing Precisions

**Figure 8:**
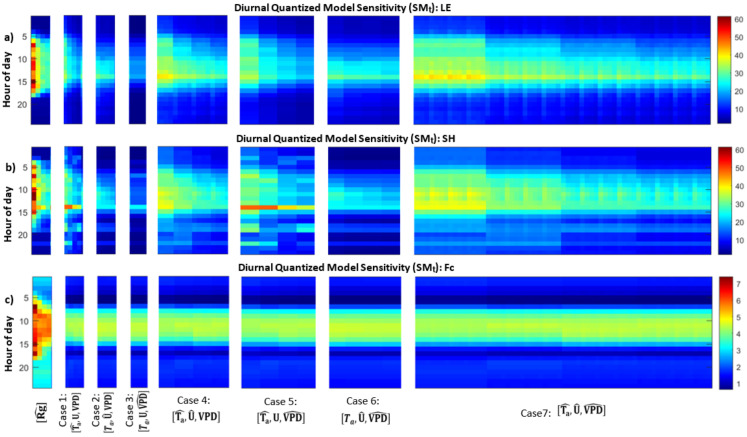
Diurnal quantized model sensitivity (SM_t_) for: (**a**) *LE* (W/m${}^{2}$); (**b**) *SH* (W/m${}^{2}$); and (**c**) *Fc* (μmol/m${}^{2}s$) for all cases (Equation (6)). Red colors indicate high sensitivities to quantization.

Next we explore the range of cases for which individual forcing variables and combinations of forcing variables are quantized to different extents. Specifically, we explore how quantizations of meteorological input combinations influence *LE*, *SH*, and *Fc* differently at different times of day (Figure 8a–c). First, we note a transition early in the morning where the quantized models switch from low to high sensitivity relative to the full model. SM_t_ is higher during the day for all fluxes, which is associated with higher flux magnitudes during the day. We previously noted that the effect on *LE* due to quantization of *Ta* is actually disproportionately higher at night when flux magnitude is taken into account. This effect of higher flux magnitude is also true for the effect of *U* on *LE*, but not for *VPD* on *LE* or for the effect of both (*U* and *VPD*) on *SH* (Figure 8a,b, Case 2 and Case 3). Otherwise, the effect of quantization varies over inputs and times of day for each model case. For *LE* and *SH* (Figure 8a,b), we see that the cases for which *Rg* and *Ta* are quantized (Case Rg, Case 1, and portions of Cases 4, 5, and 7) particularly lead to more sensitivity to quantization. Meanwhile, quantizing *Ta* influences *SH* more than *LE* (Figure 8a,b, Case 1). *SH* results show a less smooth pattern relative to *LE* over the diurnal cycle. *LE* is constrained by stomatal conductance and water availability in addition to temperature, and we see changes in *Ta* have an effect where the diurnal cycle dominates. However, since *SH* is more directly associated with *Ta* and *Rg* changes, we see quantization has a more varied effect with levels of quantization and times of day.

*LE* is responsive to small fluctuations in wind speed (*U*) relative to *SH*, as shown by the pattern in Case 2 with increasing levels of quantization (Figure 8a,b). In the model, *U* drives leaf and soil evaporation, and we find that *LE* is more sensitive to all levels of precision changes in wind speed, while *SH* has an increasing level of sensitivity as *U* is quantized from two to five levels. In other words, *LE* is more responsive to any changes in *U* precision during the day, while *SH* is less responsive unless *U* is quantized to very low precision. We can see the same pattern in other cases where *U* is one of the quantizing variables (Case 4, Case 6, Case 7). Meanwhile for Case 3 where *VPD* is quantized, the model is not highly sensitive to quantization, and *LE* and *SH* have similar responses for all levels of quantization.

For *Fc*, we see that all quantized model cases are nearly identical for combinations of *Ta*, *U*, and *VPD* (Figure 8c). All quantized forcing cases lead to a clear diurnal pattern in *Fc*, indicating that any quantization of inputs has an influence that varies by time of day. Since the net leaf carbon dioxide uptake (*An*) is a strong function of *PAR* (photosynthetically active radiation) and *PAR* is computed based on *Rg*, *Fc* is most strongly affected by quantizing *Rg*, and we see a clear pattern in which the effect decreases for more levels of *Rg* quantization. For other forcing variables, the lack of change with different *N* levels indicates that *Fc* is sensitive to the smallest variations in forcing data that are omitted through the quantization process, regardless of whether a forcing variable is quantized into N=2 or N=5 categories. Since leaf photosynthesis depends on effects of *PAR*, leaf temperature, and $CO_{2}$ concentration in the intercellular air space, *Fc* is sensitive to other variables more indirectly. In general, the forcing data get filtered through an iterative process to calculate stomatal conductance, leaf carbon, and leaf temperature [40,41], and we see a minimal level of sensitivity to any change in weather conditions.

In this analysis, we find that each estimated flux responds differently to the same changes in precision of input variables, indicating various levels of sensitivity due to the different mechanisms that relate model inputs to outputs. Model changes due to quantizing *Rg* tend to always be larger than other quantized cases, which indicates that the energy balance has a large influence relative to other meteorological forcing variables (*Ta*, *U*, and *VPD*) in the MLCan model even when those variables are quantized in combinations.

### 3.3. Forcing Complexity and Model Behaviors

**Figure 9:**
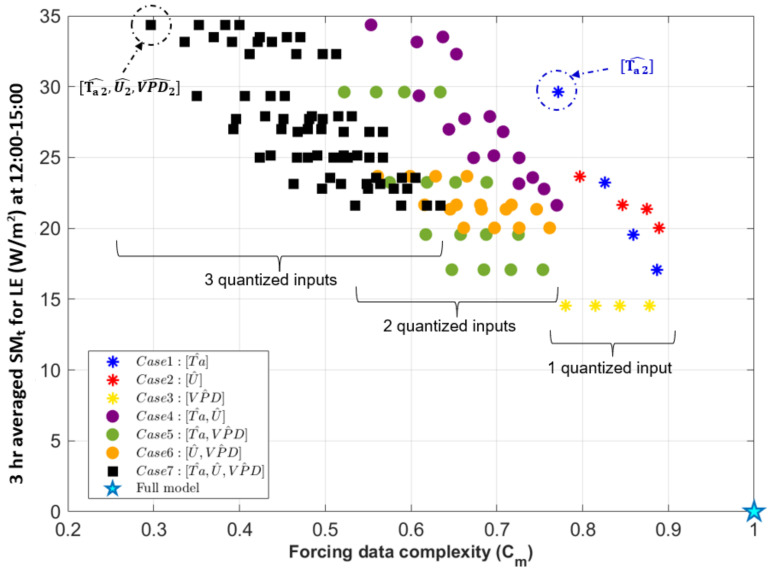
Comparison between the model and all quantized cases for (N∈[2,3,4,5]) based on their forcing data complexity, $C_{m}$, and 3-h averaged modeled diurnal sensitivity, SM_t_, at 12:00–15:00 for *LE*. The cyan star indicates the full model case ($C_{m}=1$ and SM_t_ =0). Asterisks indicate individual quantized variables (Case 1–3, 4 model runs for each case), circles indicate jointly quantized variables (Cases 4–6, 16 model runs for each case), and black squares indicate Case 7 where all three variables are quantized and there are 64 individual model runs.

Here we compare quantized model sensitivity relative to the full model (SM_t_, Equation (6)) with forcing complexity ($C_{m}$, Equation (7). We first consider $C_{m}$ and 3-h averaged modeled diurnal cycle sensitivity for all seven quantized cases between 12:00–15:00 (Figure 9). In general, decreasing forcing data complexity for all quantized cases leads to larger differences between the quantized and the full model. For example, from Figure 9 we see that a 50% change in $C_{m}$ leads to about a 20–30 W/m${}^{2}$ change in SM_t_. Quantization of any input variable to N=5 levels or fewer will result in at least 15 W/m${}^{2}$ of change in the modeled *LE* (blank area in lower right corner of Figure 9). We also detect general behaviors related to each individual input variable as follows:

- Quantized *VPD* (yellow asterisks in Figure 9) shows a horizontal pattern in the ($C_{m}$, SM_t_) space, which means quantization decreases forcing data complexity, but different levels of quantization (between N= 2 and 5) do not affect model output for *LE*. This indicates that the modeled *LE* is somewhat responsive to a change away from the instrument precision of *VPD* but does not vary further as the precision *VPD* is decreased even more. In general, this forcing variable can be greatly simplified without changes in model sensitivity. Moreover, quantizing *VPD* leads to the lowest SM_t_ indicating the lowest sensitivity at this time of day of Cases 1-3, for which a single variable is quantized.
- Quantized *Ta* (blue asterisks in Figure 9) similarly decreases forcing data complexity but results in different model behavior based on each level of quantization. For example, the N=2 scenario of Case 1 has the largest *LE* sensitivity among other individually quantized forcing variables at this time of day. This also translates to other quantized cases where *Ta* is quantized to N=2 levels (Case 4, 5, 7) that lead to higher model sensitivity.
- Finally, quantizing *U* (red asterisks in Figure 9) also influences both model behavior and forcing complexity. Quantization decreases forcing data complexity similarly, but model sensitivity is less than when *Ta* is quantized.

Cases 4-7 for which two or three forcing variables are quantized (circles and squares in Figure 9) represent different combinations of quantized variables and show different and more variable patterns. As expected, $C_{m}$ decreases as more variables are quantized, and the lowest $C_{m}$ is a subset of Case 7 for which all three inputs are quantized to N=2. In Case 4, there is a wide range of differences in SM_t_ in which *Ta* and *U* are quantized (purple circles in Figure 9). In Case 5, for which *Ta* and *VPD* are quantized (green circles in Figure 9), the tightly clustered subsets along the $C_{m}$ axis are associated with quantized *VPD*, and the differences in SM_t_ are mainly attributable to different quantization levels for *Ta*. This follows the previous finding that quantizing *VPD* on its own (Case 3) does not lead to changes in model behavior. In Case 6, in which *U* and *VPD* are quantized (orange circles in Figure 9), while there is a similar horizontal pattern for changes in *VPD* quantization levels, smaller differences in SM_t_ and $C_{m}$ between groupings are due to quantizing *U*.

Case 7 ($\hat{\mathrm{Ta}},\hat{U},\hat{\mathrm{VPD}}$) illustrates that quantizing different forcing variables simultaneously can have a nonlinear impact in terms of both joint entropy of the forcing data and model performance measures. For this case (black squares in Figure 9), there are clusters of models with similar SM_t_ and differences in $C_{m}$ in each cluster. This indicates that there are groupings of model inputs for which the forcing complexity can be greatly decreased without changing model sensitivity, but behavior depends on which forcing variable is altered. Namely, for *LE* the differences between clusters are associated with changing the precision of *Ta* as determined from the other cases.

**Figure 10:**
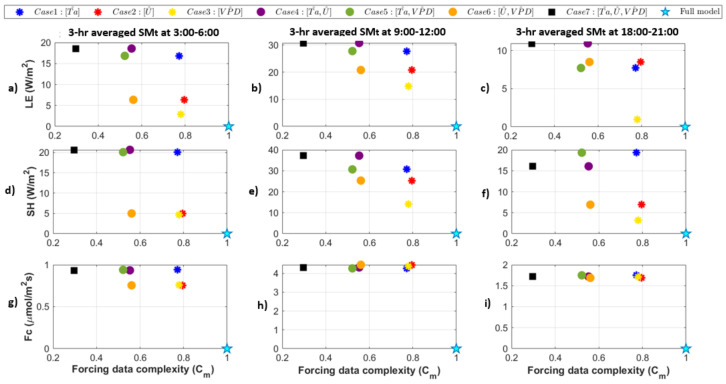
Comparison between full model and quantized cases ($N_{T}=2$, $N_{U}=2$ and $N_{VPD}=2$ levels) based on their forcing data complexity and 3-h averaged model diurnal SM_t_ for *LE* (**a**–**c**), *SH* (**d**–**f**), and *Fc* (**g**–**i**) for three times of day. Left panels (**a**,**d**,**g**) indicate all 7 quantized model behaviors at 3:00–6:00 am. Middle panels (**b**,**e**,**h**) indicate all 7 quantized model behaviors at 9:00–12:00. Right panels (**c**,**f**,**i**) indicate model behaviors at 18:00–21:00.

Quantized model behavior varies for different model output variables and different time points. This is evident when we compare between *LE*, *SH*, and *Fc* for three time ranges, at 3:00–6:00, 9:00–12:00, and 18:00–21:00 (Figure 10). Here, we only show one quantized model run for each case and choose the N=2 quantization which corresponds to the lowest $C_{m}$ within a given case. For example, the Case 7 point corresponds to the model forcing where all three input variables are quantized to N=2. In these examples, Case 7 ($\hat{\mathrm{Ta}},\hat{U},\hat{\mathrm{VPD}}$) is the case that most minimizes the complexity of the forcing dataset. This is a subset of the full suite of model forcing complexity but shows the most extreme changes in both forcing complexity and model behaviors. We found that there is a linear relationship between $C_{m}$ and SM_t_ during the day for the heat fluxes (Figure 9b,e), where the lowest precision cases lead to a large reduction in complexity and tend to have the most impact on modeled fluxes. In other words, there is an approximate line that relates $C_{m}$ to SM_t_ during the day (middle panels) even though $C_{m}$ reflects a different combination of forcing variables that are manipulated. Meanwhile for early morning and evening time ranges, the relationship between forcing complexity and model behavior is more related to whether air temperature (*Ta*) is quantized or not.

We also find that quantization to N=2 levels results in different thresholds in terms of the minimum difference between an original and quantized model result. For example, for Figure 9, the gap between full model and quantized model results is about 15 W/m${}^{2}$ for *LE* at 12:00–15:00. However, in Figure 10, it is much smaller for 3:00–6:00 and 18:00–21:00. Particularly Case 3 (the yellow asterisk in Figure 10c) has very low sensitivity to *VPD* at that time of day relative to other combinations of cases that have large effects up to 10 W/m${}^{2}$ even during the nighttime. Here we could draw two separate “best fit” lines, one for “*Ta* is quantized” and one for “*Ta* is not quantized”, since the model sensitivity to any other input is relatively low. We see cases where *U* and *VPD* quantized individually or jointly have the lowest SM_t_ during the nighttimes, indicating the model is particularly unresponsive to the precision of those variables. This can be explained in that *U* and *VPD* are typically low and relatively stable during the night, so quantizing has less effect on model results. Finally, for *Fc*, the gap from the model to the quantized cases is always the same (Figure 10g–i). This indicates that *Fc* has a response to quantization with a different threshold than the other fluxes. For example, *Fc* may show a more variable response for quantization to many higher levels which are not considered here.

## 4. Discussion and Conclusions

Forcing uncertainty is an important aspect in model development, and this study illustrates a variety of effects of forcing precision, which can be determined by measurement devices or data storage, on model behaviors. Particularly, quantization of forcing data makes it possible to isolate model sensitivity to different ranges of variability of forcing variables, and the Lloyd algorithm enables us to optimally quantize variables to retain maximum information relative to the original distribution. We performed several quantization experiments using MLCan, a complex ecohydrological model with multiple high-resolution forcing variables and many simulated processes ranging from the canopy radiation regime to heat and carbon fluxes from soil and canopy subsystems. While quantizing incoming solar radiation had a large and relatively predictable effect as a control on the model energy balance, quantizing meteorological variables caused more nuanced model responses. We summarize these experiments in the context of specific MLCan results as follows:

- **Energy balance constraints:** When incoming solar radiation is quantized, sensible and latent heat fluxes shift to meet the energy balance with some gaps that can be attributed to other energy fluxes that are not analyzed here, specifically ground heat flux and longwave radiation. Canopy carbon fluxes respond more nonlinearly and depend on whether the quantization increases or decreases the apparent radiation.
- **Temporal variability and dependence on system states:** We next explored model effects of air temperature quantization for different growing season time windows. This highlighted the connection between model sensitivity to forcing precision and system states, such as wetness or vegetation conditions. For example, quantized air temperature most influences latent heat fluxes during a relatively hot and dry period at the end of the growing season. This connects to previous findings based on observed data that causal interactions in ecohydrologic systems vary seasonally and with wetness conditions [55].
- **Joint effects of multiple forcing precisions:** Finally, we quantized combinations of air temperature, vapor pressure deficit, and wind speed to different extents, and plotted model responses along a forcing complexity axis, where forcing complexity indicated the extent to which the joint entropy of the forcing variables was reduced. From this, we can determine which combinations of variables are “most compressible” in terms of their ability to reduce model forcing complexity and which lead to large changes in model behavior. For example, a quantization that leads to vary forcing complexity with a very small change in model behavior would indicate that the model is not sensitive to a particular input or combinations of inputs, and the model forcing or use of that input could be simplified in some way. On the other hand, this could also indicate that the model is not utilizing the full extent of available information in the forcing and could motivate other changes to the model structure. We saw a general trend in which simulated heat fluxes diverge more from the original model case as multiple variables were increasingly quantized, but there was a wide range of forcing complexities that led to the same model sensitivity. This illustrates that the effect of forcing precision varies depending on the degree of quantization (*N*) and the combination of variables involved. In other words, it is possible to determine a set of forcing variables that can be maximally simplified with minimal change in model behavior.

Overall, MLCan heat and carbon fluxes were relatively insensitive to model meteorological forcing precision in those results of quantized inputs for air temperature, vapor pressure deficit, and wind speed. This insensitivity indicates that the quantization of those forcing variables cannot greatly improve or worsen modeled fluxes. This lack of sensitivity is due to both the complexity of the MLCan model, since there are many inputs, processes, and parameters that contribute to model behavior [11,40] and the quantization algorithm that is set up to act as an optimal representation of the original data. Overall, our results indicate that MLCan relies on representations of the general magnitudes of meteorological forcing data, and the model structure does not take advantage of information contained in the variability of individual variables.

While the results presented here are specific to a particular model and study site, we expect they likely reflect a variety of process-based ecohydrologic modeling frameworks that use similar process sub-models and numerical schemes. Meanwhile, the method of forcing quantization could be used in conjunction with other individual or multiple working hypothesis (parameters sets, model structure, and process equations) to complement a wider sensitivity analysis and to better diagnose complex model uncertainty. For example, numerical solver methods and multiple representation models of hydrologic processes (e.g., Structure for Unifying Multiple Modeling Alternatives (SUMMA) [56]) could be compared in terms of their sensitivity to forcing precision. Similarly, we could compare different modeling frameworks, such as the Joint UK Land Environment Simulator (JULES) and the Noah-MP models, which support different options for a range of biophysical and hydrologic processes [57,58]. Further extensions of this work could address how model complexity, in terms of the number of input variables, parameters, or other aspects, leads to different sensitivities to forcing. For example, we would expect a very simple model with few input variables to be more highly sensitive to changes in those input variables. For these types of models, methods such as the Lloyd algorithm for rate-distortion theory could have a larger impact in that it is more important to obtain an optimal representation of forcing data.

The methods presented here could be used in tandem with global sensitivity analysis (GSA) methods that seek to determine how ranges of parameters have a joint effect on model output [59]. For example, the impact of forcing precision could be compared with global sensitivity to a given forcing variable [19], detected interactions between model input variables [60], or correlated input factors based on Latin hypercube sampling (rLHS) [61]. It has previously been found that different GSA methods can provide different rankings of parameters, and no single GSA method can be said to comprehensively identify the most important or relevant features of models [62]. With this, the integration of forcing temporal variability and precision as a component of model sensitivity analysis could lead to further understanding of how model parameters and forcing data interact. For example, a parameter may become more or less important in a model in which time-series input data is available at a low precision. As an extreme example, if a forcing variable was estimated as a constant (N=1 levels of quantization), it essentially becomes an input parameter, which may alter the dynamics of relevant processes.

Another application of the method proposed here is to determine the necessity for certain field or satellite measurements, given a particular model structure. For example, Raleigh [63] analyzed the effect of lacking certain forcing inputs into snowpack models that are typically unavailable at automatic weather stations and instead are often empirically estimated based on other variables or sub-models. An analysis of forcing precision could similarly be used to compare the need for certain data types or data at a certain temporal or spatial resolution. In the study presented here, a low sensitivity to meteorological forcing precision could indicate that weather data from a nearby site or satellite-derived data would be sufficient when flux tower weather station forcing is not available. In other words, additional weather stations or advanced sensors have marginal value if the model does not utilize the forcing data beyond what can be estimated from other sources at a lower precision. The range of forcing complexities explored here also illustrates the potential to greatly decrease the storage size for forcing data in certain model cases.

As models become more complex and prolific, it is important to understand detailed aspects of how different model inputs, including time-series forcing, are used within different model frameworks and for a given model under different system states. This is also relevant in the comparison of machine learning models with physically based models in that the modeling framework may dictate the extent to which a model can “recognize” the detail provided in a given forcing variable. The methods presented here could also be applied broadly beyond an ecohydrological process-based scope to better understand model behavior and the importance of obtaining input data at a given level of precision.

## Figures and Tables

**Table 1:** Specification of quantized forcing variable. The optimal bin width (*D*) is derived from $\frac{3.5\sigma }{n^{\frac{1}{3}}}$ [48], where σ is the standard deviation of the data, and *n* is the total number of data values.

Input Variable	Unit	Stdev	Opt Bin Width
*Ta*	${}^{\circ }$C	3.7878	0.5
*U*	m s${}^{-1}$	1.7032	0.3
*VPD*	kPa	0.6029	0.1
*Rg*	W/m${}^{2}$	349.02	50

## Data Availability

The MLCan2.0 model used in this research is publicly available on GitHub at https://github.com/HydroComplexity/MLCan2.0 (accessed on: 8 June 2022). Codes and the data are available on GitHub at https://github.com/allisongoodwell/Farahani_RateDistortion_Codes_Github (accessed on: 8 June 2022).

## Abbreviations

The following abbreviations are used in this manuscript:

IT	Information Theory
SA	Sensitivity Analysis
SM	Sensitivity Metric
MLCan	Multi-Layer Canopy-soil-root
CINet	Critical Zone Interface Network
IMLCZO	Managed Landscape Critical Zone Observatory
RMSE	Root Mean Square Error
GSA	Global Sensitivity Analysis
SUMMA	Structure for Unifying Multiple Modeling Alternatives
JULES	Joint UK Land Environment Simulator
MMSE	Minimum Mean Square Error
